# A High-Throughput Colorimetric Screening Assay for Terpene Synthase Activity Based on Substrate Consumption

**DOI:** 10.1371/journal.pone.0093317

**Published:** 2014-03-28

**Authors:** Maiko Furubayashi, Mayu Ikezumi, Jun Kajiwara, Miki Iwasaki, Akira Fujii, Ling Li, Kyoichi Saito, Daisuke Umeno

**Affiliations:** 1 Department of Applied Chemistry and Biotechnology, Chiba University, Chiba, Japan; 2 Precursory Research for Embryonic Science and Technology (PRESTO), Japan Science and Technology Agency (JST), Kawaguchi, Saitama, Japan; Jacobs University Bremen, Germany

## Abstract

Terpene synthases catalyze the formation of a variety of terpene chemical structures. Systematic mutagenesis studies have been effective in providing insights into the characteristic and complex mechanisms of C-C bond formations and in exploring the enzymatic potential for inventing new chemical structures. In addition, there is growing demand to increase terpene synthase activity in heterologous hosts, given the maturation of metabolic engineering and host breeding for terpenoid synthesis. We have developed a simple screening method for the cellular activities of terpene synthases by scoring their substrate consumption based on the color loss of the cell harboring carotenoid pathways. We demonstrate that this method can be used to detect activities of various terpene synthase or prenyltransferase genes in a high-throughput manner, irrespective of the product type, enabling the mutation analysis and directed evolution of terpene synthases. We also report the possibility for substrate-specific screening system of terpene synthases by taking advantage of the substrate-size specificity of C_30_ and C_40_ carotenoid pathways.

## Introduction

Terpenoids form one of the largest groups of natural products produced by plants and bacteria, and they include huge structural diversity and molecular functions including pharmaceuticals, agrochemicals, fragrances, synthetic rubber and diesel/jet fuels. Integrated efforts by synthetic biologists have dramatically improved the production levels of valuable terpenoids in heterologous hosts such as microbes or plants [1]–[5]. This improvement has primarily been achieved by metabolic engineering efforts, reactor designs, and host breeding but not by engineering the catalytic capacity of terpene synthases (TPSs). TPSs are generally slow enzymes (*k_cat_* values typically range from 1 min^−1^ to 1 sec^−1^), which is a likely general feature of secondary metabolic enzymes [6], [7]. Given the maturation of modern metabolic engineering, the major limiting factor in the total production levels of terpene compounds has been identified as the TPSs [8]. However, because of the lack of reliable screening/selection systems for TPSs, forward engineering of TPSs has been severely limited: both substrates and products of TPSs are colorless, diverse in structure, and volatile. A few investigators have used protein solubility screening [9] or proteolysis resistance [10] for TPSs but not for activity screening.

In addition, TPSs themselves have long attracted substantial interest because of their unique catalytic mechanisms [11]–[15]. Site-directed mutagenesis approaches have been used to elucidate the mechanisms of the reaction and specificity control of this enzyme [16]–[20]. Recently, some researchers conducted extensive mutagenesis on TPSs [9], [21], revealing the remarkable plasticity of this class of enzymes. At present, these massive mutagenesis studies have been performed on the basis of sequence comparisons or structural information, thereby focusing on the selected candidate residues for systematic amino acid substitution, rather than random mutagenesis. This approach is largely explained by the lack of convenient methods for the direct screening/selecting for TPS activities.

A systematic and high-throughput screening of TPS activities should greatly accelerate the advancement of the two fields indicated above. To this end, Lauchli et al [22] demonstrated that TPS could be a target for directed evolution by using a surrogate substrate for FPP. They synthesized vinyl ether containing isoprenyl diphosphate, which is similar to FPP. The conversion of this molecule releases methanol, which can be enzymatically visualized, and provides a basis for evaluating TPS activity. Withers et al. [23] employed the inherent toxicity of prenyl diphosphate precursor molecules to terpenes, which could be mitigated by TPS activity. A careful selection of growth media enabled the selective growth of cells harboring genes that encode active TPSs, which convert isoprenyl diphosphates into non-toxic terpene compounds. Theoretically, this strategy should enable a selection for any type of TPS and/or isoprenyl transferase, irrespective of their product types, as long as they consume the precursors.

**Figure 1:**
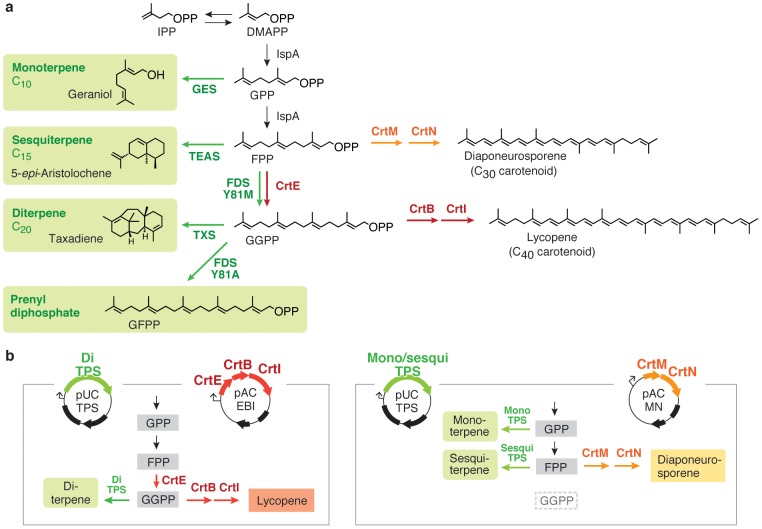
Carotenoid pathways compete with TPSs for diphosphate precursors. (**a**) *E. coli* biosynthesizes GPP and FPP using endogenous farnesyl diphosphate (IspA), which provides direct substrates for monoterpene and sesquiterpene synthesis, respectively. Using FPP as a starter molecule, *S. aureus* CrtM and CrtN biosynthesize diaponeurosporene, a yellow C_30_ carotenoid. By expressing GGPP synthase (CrtE from *P. ananatis* or *Pantoea agglomerans* in this work), a large portion of endogenous FPP is converted to GGPP. GGPP can be fed either to diTPSs or to the pathway of C_40_ carotenoid pigment lycopene (by CrtB, and CrtI from *P. ananatis* or *P. agglomerans*). Geraniol synthase (GES) from sweet basil, 5-*epi*-aristolochene synthase (TEAS) from tobacco, and taxadiene synthase (TXS) from Pacific yew were used to represent monoTPSs, sesquiTPSs, and diTPSs, respectively. *G. stearothermophilus* farnesyl diphosphate synthase (FDS) and its specificity-shifted variants (FDS_Y81A_ and FDS_Y81M_) [41] were also tested. (**b**) Left panel: diTPS competes for GGPP with C_40_ carotenoid enzymes. pAC-EBI harbors *crtE*, *crtB* and *crtI* genes from *P. ananatis* under the control of the *lac* promoter. Right panel: monoTPS or sesquiTPS competes for FPP with C_30_ carotenoid enzymes. pAC-MN harbors *crtM* and *crtN* genes from *S. aureus* under the control of the *lac* promoter.

For this study, we developed a high-throughput colorimetric assay for TPS based on substrate consumption. This assay is based on the fact that the carotenoid pigments, another important subgroup of isoprenoid compounds, uses the same isoprenyl diphosphates (FPP and GGPP) as substrates (Fig. 1); here, the expression of active TPSs results in decreased availability of building blocks for carotenoid biosynthesis, thereby reducing the pigmentation of the host cells. In theory, the activity of any TPS can be rapidly detected by assessing the color intensity of the colonies. Using this method, we could visualize the landscape of the mutant libraries, rapidly isolate the active terpenoid genes from inactive ones, and search the variants for improved activities. In addition, by taking advantage of the substrate-size specificity of carotenoid pathways, we explored the possibility of using substrate-size-specific screening for monoterpene synthase (monoTPS), sesquiterpene synthase (sesquiTPS) and diterpene synthase (diTPS) activities.

## Results

### Construction of screening plasmids

The proposed screening method is simply performed by co-expressing TPS genes in a carotenoid-producing cell. Here, the TPSs and carotenoid pathways compete for the same isoprenoid precursors (Fig. 1); when the TPSs are sufficiently active, their expression deprives the substrate of carotenoids, resulting in a reduction in cellular pigmentation. The more active the TPSs, the lower the cellular color will be. This trend allows for the visual high-throughput detection of TPS activity simply by using the colony hue.

The substrate for most of the natural carotenoids is geranylgeranyl diphosphate (GGPP). Three genes must be exogenously expressed for the biosynthesis of lycopene in *E. coli*. We constructed pAC-EBI (Fig. 1), a plasmid containing *crtE*, *crtB*, and *crtI* genes from *Pantoea ananatis* under a *lac* promoter. CrtE (GGPP synthase) provides GGPP, a substrate for phytoene synthesis catalyzed by CrtB (phytoene synthase). CrtI (phytoene desaturase) desaturates phytoene into the red pigment lycopene. We expected to use this plasmid primarily to detect/score the activity of GGPP consumers such as diTPSs, which directly compete with CrtB for GGPP.

We also constructed a plasmid pAC-MN to express *Staphylococcus aureus crtM* and *crtN*, for producing C_30_ carotenoid pigments. CrtM (diapophytoene synthase) selectively uses FPP to construct diapophytoene, which is then desaturated by CrtN (diapophytoene desaturase) to yield diaponeurosporene yellow pigments (Fig. 1) [24], [25]. The diTPSs activity cannot influence the pigment synthesis by this plasmid because GGPP substrate is not present in the cell. We expected this plasmid to be useful primarily for screening sesquiTPSs, which directly compete with CrtM for FPP. In theory, monoTPS could reduce the pigment production of this cell by capturing GPP, the intermediate released by endogenous GPP/FPP synthase (IspA).

During the process of constructing this plasmid, it happened to acquire an *E. coli* insertion sequence (IS10) between the *lac* promoter and the *crtM* gene. Interestingly, we found that this acquisition improved the robustness of the screening, and we attempted to remove the IS10 sequence, resulting in a severe reduction in transformation efficiency. Therefore, we decided to adopt the plasmid that contained an IS10 sequence (The sequence is detailed in Text S1).

### Visualizing the cellular activities of diTPS

**Figure 2:**
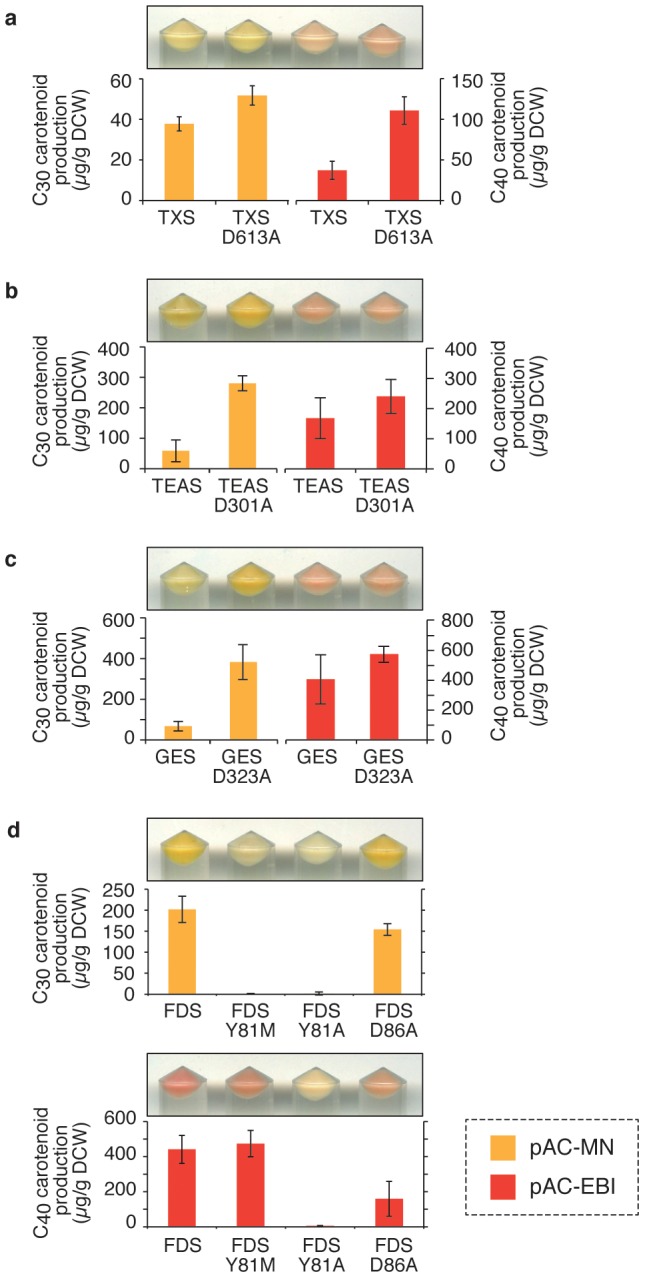
The effects of the expression of terpene synthase and prenyltransferase genes on the production level of C_30_ and C_40_- carotenoids in *E. coli*. The TPSs on pUC18m vectors were expressed in *E. coli* strain XL1-Blue harboring pAC-MN (yellow bars) or pAC-EBI (red bars) with TXS variants (**a**), TEAS variants (**b**), GES variants (**c**), and FDS variants (**d**). After 48 hours of culture, carotenoid production was analyzed by extracting the carotenoid pigments with acetone and measuring the absorbance. The bars indicate the average of four samples, and the error bars indicate the standard deviation. Cell pellets before acetone extraction are shown above each of the bars.

First, we tested taxadiene synthase (TXS), a diTPS from Pacific yew [26], [27], the first committing enzyme in taxol biosynthesis. As an active TXS, we constructed TXS-M60, a TXS gene starting from the 60^th^ residue with a truncated N-terminal signal peptide [27]. As an inactive variant, we constructed TXS-M60_D613A_ by substituting the first Asp residue of the conserved metal-binding (D^613^DXXD) motif required for catalysis (loss of activity confirmed: Fig. S1). The co-expression of TXS-M60 with pAC-EBI resulted in reduced cellular color and in pigment production (Fig. 2a). No such decrease was observed for the inactive variant TXS-M60_D613A_. Thus, cellular activity of TXS was readily detected as the reduction in cellular pigmentation. We also observed a similar result by using another lycopene-producing plasmid, pAC-LYC [28] (Fig. S5). Unlike in pAC-EBI or pAC-LYC, the co-expression of TXS-M60 with pAC-MN only resulted a very small decrease in the pigmentation (Fig. 2a) when compared with inactive variant TXS-M60_D613A_. TXS reportedly has promiscuous activities as sesquiTPS [29]; this finding might explain the slight decrease in C_30_ carotenoid pigmentation.

### Visualizing the cellular activities of sesquiTPS

Tobacco 5-*epi*-aristolochene synthase (TEAS) is one of the most extensively studied plant sesquiTPSs [30], [31]. When wild-type TEAS was co-expressed with pAC-MN, we observed a considerable decrease in the C_30_ carotenoid pigment (Fig. 2b). By contrast, an inactive variant of TEAS_D301A_, which was created by removing the conserved metal-binding (D^301^DXXD) motif required for catalysis (loss of activity confirmed, Fig. S2), did not cause this effect. This finding clearly indicates that the consumption of FPP to produce 5EA is the direct cause of the decreased pigmentation. The expression of TEAS and its variants showed almost no effect on the pigmentation of cells harboring pAC-EBI. This result shows that TEAS does not deprive FPP of the C_40_ carotenoid pathway. Considering that the prenyltransferases have a much high turnover rate (*k_cat_*∼1 sec^−1^, for example, ref. [32]–[34]) than that of TEAS (0.04 sec^−1^) [35], it is conceivable that CrtE, a GGPP synthase, was efficient enough to convert the available FPP into GGPP and was unaffected by the co-existence of TEAS. Note that most (if not all) reported sesquiTPSs have *k_cat_* values similar to that of TEAS (for example, see [36]–[38]).

### Visualizing the cellular activities of monoTPS

Other important isoprenoid members are the monoterpenes (C_10_ terpenes), which use GPP as a substrate. By co-expressing active geraniol synthase (GES) from sweet basil [39] with pAC-MN, we observed a significant decrease in the C_30_ carotenoid pigment (Fig. 2c). This color reduction was not observed for the inactivated variant GES_D323A_. Because geraniol is the only product detected when GES is expressed in *E. coli*, and there was no signal for related sesquiterpenes such as farnesol (Fig. S3), it is unlikely that GES is directly competing with CrtM for FPP. This finding is consistent with other reports about GES expression in *E. coli* (for example, ref. [39]). The prenylation of tRNAs [40] is known to be fully functional without a specialized GPP synthase enzyme in *E. coli*, and overexpressing monoTPSs results in a significant production of monoterpenes by *E. coli* [39]. Thus, endogenous GPP/FPP synthase (IspA), which is a two-step enzyme (Fig. 1), seems to release its intermediate GPP in a capturable format. As a result, the C_30_ carotenoid pigments can be significantly reduced not only by sesquiTPSs but also by monoTPSs, enabling high-throughput screening for this class of enzymes as well.

### Discriminating between the FPP and GGPP consumption activities of prenyltransferase

To test whether this screening method can be used for other enzymes that also use isoprenyl diphosphates as substrates, we tested the effects of expressing size-specificity *Geobacillus stearothermophilus* mutants of farnesyl diphosphate synthase (FDS) on carotenoid production. The product size specificity of FDS has been extensively examined by Ohnuma et al. [41]. Among the various size-specificity variants described in that work, we chose the Y81M variant of FDS (FDS_Y81M_), which exhibits very high product specificity to GGPP. We also used FDS_Y81A_, which produces geranylfarnesyl diphosphate (GFPP, C_25_) in a relatively efficient manner. When co-expressed with pAC-MN, these variants both decreased C_30_ carotenoid production to an undetectable level (Fig. 2d), indicating the consumption of FPP by both variants. By contrast, C_40_ carotenoid was only decreased when using FDS_Y81A_ (GFPP synthase) in co-expression with pAC-EBI, but it was increased when using FDS_Y81M_ (GGPP synthase). This result shows that the screening system could also be applied to prenyltransferases. It also indicates that our screening method could discriminate FPP consumers from GGPP consumers using these plasmids.

### Removing deleterious mutations from taxadiene synthase

**Figure 3:**
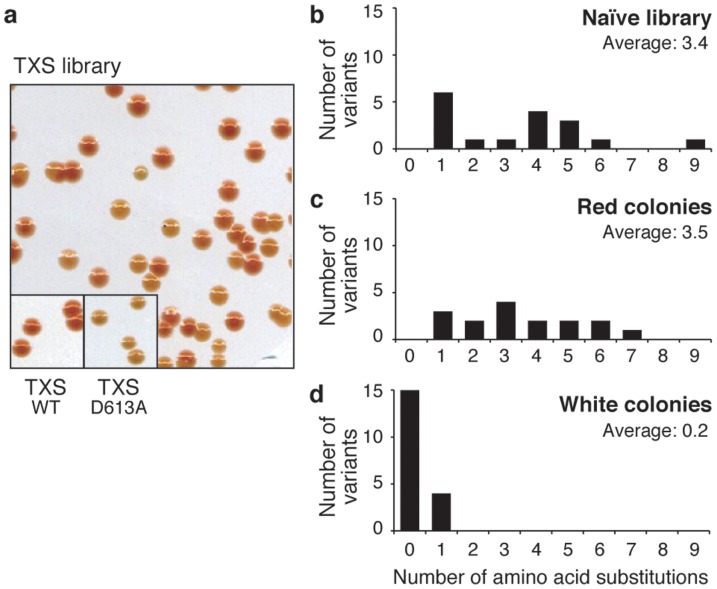
Purifying selection against deleterious mutations in TXS. (**a**) The *E. coli* colonies harboring pAC-LYC that were transformed with TXS libraries. (**b**–**d**) The distribution in the number of mutation in the randomized region (671–771 aa) of TXS variants isolated from naïve library (**b**), red colonies (**c**), or while colonies (**d**).

Removing deleterious mutations or discriminating between active/inactive mutations is important in the forward engineering or mutation analysis of given enzymes. We investigated how effectively our method can remove inactivating mutations from variant pools of TPSs. Random mutations were introduced into a part of active site domain (671–771th residues) of TXS using error-prone PCR. The resulting TXS library was cloned into the vector under the control of an *araBAD* promoter, and it was then transformed into *E. coli* XL1-Blue cells harboring pAC-LYC. The transformant colonies could be divided into the following two groups: ones with a red color and others with a pale (beige) color. Several clones, both from red or beige colonies, were randomly picked for sequence analysis. The average number of mutations was 3.5 in the variants from red colonies, and it was close to that found in the naïve library (before selection) (Fig. 3). By contrast, most of the variants from pale colonies showed no amino acid substitution, and only 1 substitution was found in 4 variants. Conversely, the 53 amino acid substitutions, together with 1 frameshift mutation and 2 stop codons, were found in 16 variants that were taken from the red colonies. All of these results indicate that deleterious or negative mutations were effectively removed from the pool simply by picking the pale colonies.

### Correlating pigmentation levels with TPS activity

In theory, TPSs with higher activities should result in a higher level of substrate deprivation from the carotenoid pathway, thereby resulting in a lower level of pigmentation. If this assumption is true, correlation between color formation and enzyme activity can be used to not just discriminating active/inactive TPSs; it can be used to truly ‘score’ or ‘rank’ the cellular activity of TPSs.

**Figure 4:**
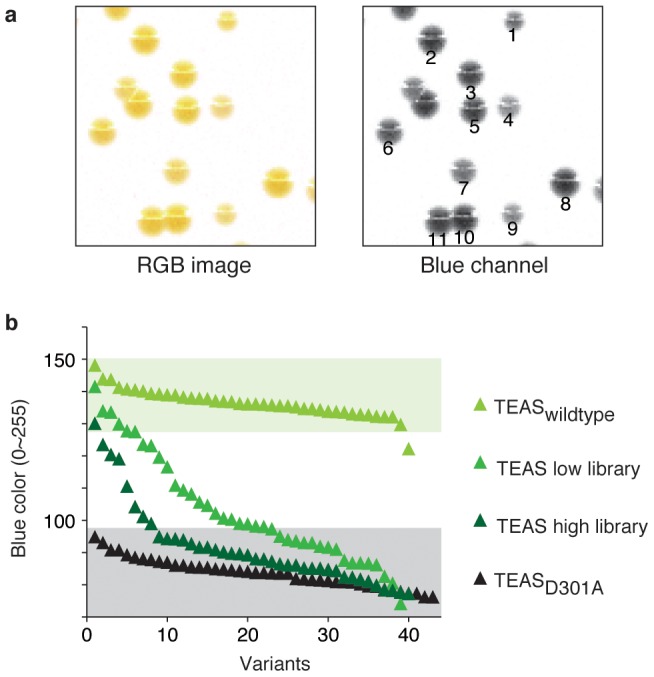
Fitness landscape of the TEAS variant pools. (**a**) *E. coli* colonies harboring pAC-MN and TEAS variants (representative colonies from the TEAS low library are shown). The cells were plated on LB-agar topped with a nitrocellulose membrane to provide a white background for demonstrating the colony color. The left panel shows the raw image taken by the image scanner. This image was subdivided by the RGB channels, and the blue channel shown in the right panel was used directly to represent the “yellowness” of the colonies. (**b**) Fitness landscape drawn using the values from the blue channel intensity. The image at the right of panel **a** was analyzed using ImageJ. The grey scale image in 8 bit ranged from 0 (black) to 255 (white), in which the whiter colonies will show the higher scores. The ranges we defined as “wildtype-level” and “dead” are indicated in green and grey background, respectively.

We explored this possibility using a mutant TEAS library. We constructed two mutant TEAS libraries with high and low mutation rates by error-prone PCR under two different reaction conditions. These libraries were introduced into *E. coli* XL1-Blue cells harboring pAC-MN, and they were then plated onto an agar plate topped with a nitrocellulose membrane to provide a white background for the colonies. Color pictures of the resulting colonies were recorded using a tabletop scanner. The pigmentation levels of the colonies were individually scored by ranking their ‘yellowness’ by dividing the scanned image by the RGB channels from which the intensity value of the blue channel was extracted (Fig. 4a). Each of the colonies was independently scored by its average darkness. Thus, we could rank hundreds of clones based on the color of their colonies. While both the wild-type TEAS and the inactive mutant (D301A) yielded a narrow range of scores, the mutant libraries exhibited a wide distribution in scores. Sorting by Blue value scores yielded fitness landscapes (Fig. 4b), which are typical for random libraries. The high library gave steeper landscape with larger fraction of ‘dead’ mutants.

### Directed evolution of TEAS

We searched for mutations that improved the cellular activity of TEAS. The two TEAS libraries in the previous section were transformed into *E. coli* XL1-Blue cells harboring pAC-MN. Following the formation of visible colonies on LB-agar after 24 h of incubation at 37°C, the plate was placed at room temperature (20–25°C) for an additional 24–48 h. From *ca*. 2000 colonies screened from two libraries, 42 colonies (25 and 17 from low or high libraries, respectively) were whiter in color than those harboring wild type TEAS. After re-screening them, we extracted plasmids from 30 clones that were especially whiter in color and analyzed their sequences.

Many variants possessed mutations in the first 35 nucleotides of the TEAS gene (Table S1). We speculate that these mutations increased the translation efficiency by destabilizing the mRNA structure; the translation efficiency calculated by an RBS calculator [42] was found to be higher in most of these variants (Table S1) than that of their parent (wildtype TEAS). Among the 30 analyzed, 7 variants were free from mutations in the first 35 nucleotides on the 5′-end of PCR-amplified regions. They might be those with higher catalytic efficiency, higher substrate affinity, or higher stability inside the cell. We re-transformed *E. coli* XL1-Blue cells harboring pAC-MN with these seven variants, and we reconfirmed that the colony color was paler than that of the wild type TEAS (Fig. S4). Two variants called TEASmut7 and TEASmut11, which exhibited the lowest pigmentation (or highest B-values), were found to have single amino acid substitutions at Q481R and L399R, respectively (Table S2).

**Figure 5:**
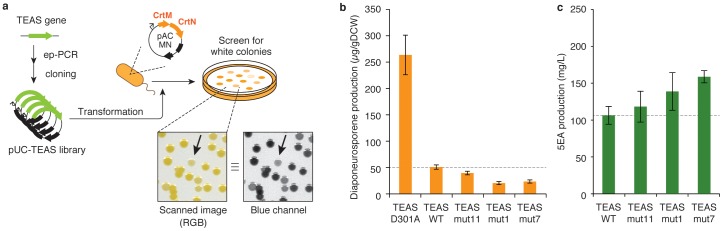
Directed evolution of TEAS. (**a**) Procedure for the directed evolution of TEAS for higher cellular activity. (**b**) The carotenoid production level of *E. coli* cells harboring TEAS variants and pAC-MN-idi. (**c**) *E. coli* production of 5EA by TEAS variants. FDS and Idi are additionally and constitutively expressed.

In addition to TEASmut7 and TEASmut11, we selected one more variant TEASmut1 with single mutations in N-terminal domain, which seemed to have higher translation efficiency (in the RBS score) (Table S1). We transformed these variants into *E. coli* XL1-Blue cells harboring pAC-MN-idi. The expression of wild-type TEAS resulted in the production of 50 μg/g DCW carotenoid pigments, which is one-fifth of the value found in cells that expressed the inactivated mutant TEAS (Fig. 5b). All of the TEAS mutants obtained above yielded a lower (20–40 μg/g DCW) level of pigment production. These mutants were then co-transformed with pAC-*fds-idi*, the plasmid for elevating the FPP supply (Idi: isopentenyl diphosphate isomerase), into *E. coli* XL1-Blue cells, to evaluate the production capacity of 5-*epi*-aristolochene (5EA). The production level of 5EA was higher for TEAS variants when compared with the cells expressing the wild type (Fig. 5c), indicating our screening method did isolate the TEAS variants with elevated cellular activity. Because the cellular pigmentation level had already reached nearly zero, the colonies with our carotenoid-producing plasmids were quite pale. Additional rounds of directed evolution could be achieved by tuning the screening constructs (specifically by decreasing the expression levels of TEAS variants by changing the promoter strength and/or increasing the precursor level by overexpressing upstream enzymes).

### Screening for truncation points in GES

**Figure 6:**
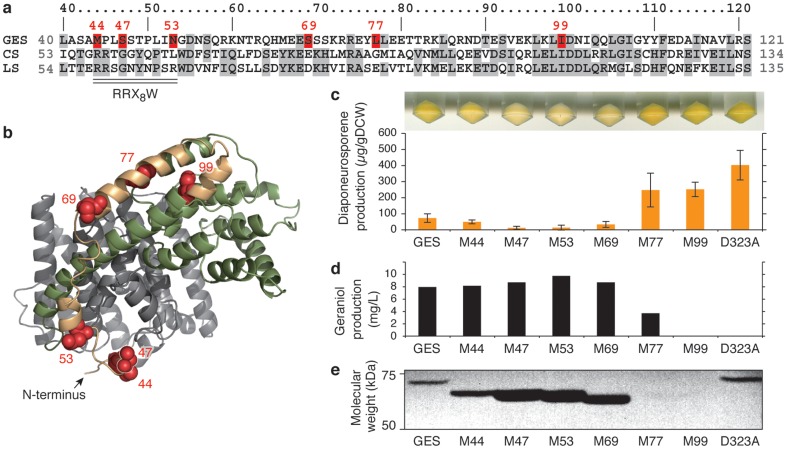
N-terminal truncation of geraniol synthase. (**a**) N-terminal alignment of GES with spearmint 4*S*-limonene synthase (LS) and sage 1,8-cineole synthase (CS), as redrawn from Iijima et al. [39]. The residues of the aligned sequences are numbered according to the residue number in the GES. The RRX_8_W motif of LS and CS is underlined. The truncated GES positions are indicated in red. (**b**) The modeled GES structure of limonene synthase (PDBID: 2ONG) by SWISS-MODEL [53]. The residues corresponding to the truncation points are indicated in red. (**c**) Carotenoid production of the cells harboring pAC-MN co-expressed with pUC-GESs. Bars indicate the average, and the error bars represent the standard deviation of four samples. The picture above the bar graph shows the colors of the cell pellets. (**d**) Geraniol production of the truncated GES variants after 8 h of culture. (**e**) Western blot of pUC-GES variants in the soluble fraction.

Plant monoTPSs are plastidic enzymes, and their N-terminal sequences are identified as plastid-targeting signal sequences [43]. The signal sequences of many plant monoTPSs are within 30–50 residues before the conserved RRX_8_W motif. Interestingly, basil GES and some other monoTPSs in the angiosperm Tps-g subfamily [44], [45] do not have this conserved RRX_8_W motif, making it difficult to predict to what extent the N-terminal sequence could possibly be truncated. Iijima et al [39] have truncated residues from the N-terminus to construct GES variants starting from Ser35 or Met44 (Fig. 6a, called S35 and M44 in this study), the N-terminal regions of the part that aligns with the RRX_8_W domain of the aligned monoTPSs. This truncated GES variant was soluble and exhibited a similar molecular weight as the plant-purified GES, but the exact truncation points in nature have not yet been elucidated.

We constructed six GES variants that were truncated at 44, 47, 53, 69, 77 and 99 and placed them under the start codon (named M44, M47, M53, M69, M77 and M99, respectively) (Fig. 6a). These variants were introduced into *E. coli* XL1-Blue cells harboring pAC-MN, and the carotenoid pigmentation level was analyzed. The cells expressing M44, M47, M53 and M69 showed decreased carotenoid production compared with those harboring inactivated GES_D323A_ or wild-type GES, indicating improved cellular activity. By contrast, the pigmentation level of M77 and M99 was as high as that for inactive mutant GES_D323A_. With respect to geraniol production, all the GES variants truncated before M69 resulted in a similar or slightly increased amount compared with the wild-type GES (Fig. 6d). M99 did not show any geraniol production, and M77 exhibited a lower but detectable level of geraniol, indicating that M77 is partially active. However, both M77 and M69 proteins did not appear in the soluble fraction (Fig. 6e). Thus, M77 and M99 are virtually non-functional or at least not suitable for practical uses in the microbial production of geraniol.

Because S35 and M44 were nearly identical in length to the plant-purified GES [39], the more truncated GES variant is not likely to be produced in nature. It is interesting, but perhaps not surprising, that GES can be truncated this much. The N-terminal RRX_8_W sequence in the other cyclic monoTPSs are needed to isomerize geranyl cations to linalyl cations [46], which are not needed for producing geraniol [39]. This might be the reason why GES could be truncated up to the 69^th^ amino acid residue. We do not know exactly why truncating at the 77/96th residue ruins the total activity of this enzyme, but it is reasonable to believe that the loss of the resultant helices (see Fig. 6b) caused GES insolubilization (Fig. 6d).

## Discussion

To continue improving the yield and efficiency of microbial terpenoid production, it is essential to increase the catalytic specification of TPSs. Directed evolution is highly suitable for identifying the mutations that increase the physical or kinetic properties of enzymes. Some TPSs had been subject to solubility screening [9] and to the phage display technique [10], both of which were used to select for the retention of physical integrity as a protein. To improve the kinetic properties (K_m_ or *k_cat_* values) of TPSs, it is desirable to directly screen for TPS activity. In addition, the massive mutagenesis programs revealed that TPSs could easily alter their reaction specificities by substituting residues that are not in contact with substrates [19], [21]. Considering that the positive mutations isolated in directed evolution of TPSs [9], [10] were all found outside the scope of *a priori* knowledge of their target enzymes, it the exploration of the entire target TPS gene should provide many more previously unexplored residues that can expand/modulate the reaction specificity of TPSs.

To date, two attempts for establishing high-throughput screening of terpene synthase activities have been published [22], [23]. A surrogate substrate was invented to visualize the TPS activity of the cell lysate [22]. This method could potentially be used for various TPSs, and there should still be some other TPSs that do not act on this artificial molecule or do not provide the desired rearrangement product. On the other hand, our carotenoid-based screening system, together with the selection system based on the mitigation of the cytotoxicity of TPS precursors [23], is based on substrate consumption rather than the detection of a specific product. Therefore, this is applicable to any TPSs irrespective of the product type. Indeed, our screening systems were applicable to all four tested TPSs and the prenyltransferases (Fig. 3), another important class of enzymes. We expect that this system would allow us to screen for various other prenyltransferases involved in quinone biosynthesis, meroterpene biosynthesis, the functionalization of tRNAs, and protein prenylation.

Our method is unique in that it could potentially be used to search for the mutations that change the substrate size specificity of TPSs. Substrate promiscuity is documented for several TPSs [29], [37], [47], but most of the known TPSs have not been tested for the possible activities for non-cognate substrates. A phylogenic analysis of plant TPSs indicates the substrate specificity diversification occurred separately after the diversification into angiosperms and gymnosperms [44], but how their specificity could have evolved remains largely unexplored. The results in Fig. 2 and Fig. 3 indicate that we could independently screen for FPP consumption and GGPP consumption using plasmids for C_30_ and C_40_ carotenoid pathways, respectively. In addition, our method clearly distinguished the size mutants of FDS (Fig. 3). As of today, we cannot discriminate between the activities of sesquiTPS and monoTPS by using the current setup with pAC-MN. By tuning the titer of GPP-to-FPP synthase activity, either by altering the size specificity of endogenous IspA or by expression balancing, it might be possible to screen these activities separately.

In addition to the screening plasmid demonstrated above, we tested several different screening constructs (for instance, Fig. S5). Most of the tested plasmids were useful for color-detecting TPS activities, and the color intensity, dynamic range, reproducibility, and detection limit of the screening were significantly affected by various conditions. Thus, the screening system must be carefully tuned so that the dynamic range of screening is appropriate to discover variants with improved activities in each given case (as discussed in Text S1). The tunable nature of this color screening is advantageous for adapting various TPSs with different specifications in a flexible manner, enabling TPSs to become routine targets of directed evolution.

## Materials and Methods

### Strains and plasmids

*E. coli* XL10-Gold Kan^R^ (Tet^r^ Δ(*mcrA*)*183* Δ(*mcrCB-hsdSMR-mrr*)*173 endA1 supE44 thi-1 recA1 gyrA96 relA1 lac* Hte [F′ *proAB lacI^q^Z*Δ*M15* Tn*10* (Tet^r^) Tn*5* (Kan^r^) Amy]) (Stratagene, CA) was used for DNA cloning and library construction, and *E. coli* XL1-Blue (*recA1 endA1 gyrA96 thi-1 hsdR17 supE44 relA1 lac* [F′ *proAB lacI^q^Z*Δ*M15* Tn*10* (Tet^r^)]) (Stratagene, La Jolla, CA) cells were used for screening and carotenoid/terpene production.

pAC-MN was constructed by amplifying the P*lac*-*crtM*-*crtN* operon from pUC-*crtM*-*crtN*, which was derived from ref. [48] and then ligated into the *Bam*HI site of pACmod. Here, pAC-MN contains an IS10 sequence between the *lac* promoter and the *crtM* gene of pAC-MN; the sequence is provided in Text S1. pAC-EBI was constructed by amplifying the P*lac*-*crtE-crtB-crtI* operon from pUC-*crtE-crtB-crtI* [48]. pAC-LYC is derived from Cunningham et al. [28].

TEAS from tobacco (*Nicotiana tabacum*) (accession no. AAA19216) that had been codon-optimized for *E. coli* were purchased from GeneArt (Regensburg, Germany). The forward primer containing an *Xba*I restriction site, a ribosome binding site, and a *Hin*dIII restriction site (5′-tttTCTAGAaggaggAAGCTTatggcatcagcagccgttgccaac-3′, restriction site capitalized and annealing site underlined) and a reverse primer with the *Xho*I site added after the stop codon were used to amplify the TEAS gene. This DNA fragment was cloned into the *Xba*I/*Xho*I pUC18m vector and named pUC-TEAS. An inactivated form of TEAS was produced by substituting an Asp 301 codon (GAT) into the Ala (GCG) codon. The his-tagged versions of these plasmids, namely pUC-hTEAS and pUC-hTEAS_D301A_, were constructed by adding a 6× His tag sequence and Gly spacer (5′-catcatcatcatcatcatggc-3′) between the start codon (ATG) and the second amino acid residue of TEAS.

TXS from Pacific yew (*Taxus brevifolia*) (accession no. AAC49310), starting from the 60^th^ residue by substituting this residue with methionine (designated as TXS-M60) and codon-optimized for *E. coli*, was purchased from GeneArt. A TXS-M60 gene was amplified with flanking *Hin*dIII and *Xho*I sites, and the PCR fragment was cloned into the *Hin*dIII/*Xho*I site of pUC-TEAS to create pUC-TXS-M60. The inactivated TXS-M60 variant was constructed by substituting an Asp 613 codon (GAC) into the Ala (GCG). The his-tagged versions, namely pUC-hTXS-M60 and pUC-hTXS-M60_D613A_, were constructed by adding a 6× His tag sequence and a Gly spacer between the start codon and the second amino acid residue of TXS-M60. pUCara-hTXS-M60 and pUCara-hTXS-M60_D613A_ (which is under the regulation of the *araBAD* promoter) were constructed by digesting the his-tagged TXS-M60 fragment at *Xba*I/*Xho*I from pUC-hTXS-M60 and pUC-hTXS-M60_D613A_, respectively, and cloned into a pUCara vector with a P*_BAD/araC_* promoter.

pUC-GES was constructed by amplifying the sweet basil (*Ocimum basilicum*) GES gene from pET-GES [39] (a gift from Prof. Pichersky) by adding an RBS/spacer sequence immediately before the ORF (5′- aggaggattacaa -3′); a 6× His tag sequence was added to the C-terminus of the gene (5′-catcatcatcatcatcat-3′) and cloned into the *Eco*RI/*Xho*I sites of pUC18m. The inactivated variant pUC-GES_D323A_ was constructed by substituting the Asp 313 codon (GAT) of pUC-GES with an Ala codon (GCG). Truncated GES variants were constructed using the primers indicated in Table S3 to PCR-amplify them using pUC-GES variants as a template and was digested/ligated by the FASTR method [49] with type IIS restriction enzyme cloning.

pUC-*fds* was constructed by amplifying the *G. stearothermophilus* FDS gene [41], [50] by adding an RBS/spacer sequence before the ORF (5′-aggaggagtaagcg-3′) and cloning it into the *Xba*I/*Xho*I restriction site of the pUC18m vector [50]. pUC-*fds_Y81A_*, pUC-*fds_Y81M_* and pUC-FDS_D86A_ were constructed by substituting the natural codon (TAC for Y81, GAT for D86) with an Ala codon (GCG).

pAC-*crtE*, which harbors a *P. ananatis crtE* gene, was constructed by deleting the *crtI* gene from pAC-*crtE*-*crtI* [50]. pAC-*fds-idi* was constructed by inserting the P*_lac_*-*fds* and P*_lac_*-*idi* fragment into the *Bam*HI and *Cla*I sites, respectively. Here, P*_lac_*-*fds* was amplified by using pUC-*fds* as a template, and P*_lac_*-*idi* was amplified from pUC-*idi*, which was constructed by inserting an *E. coli idi* gene into the *Xba*I/*Xho*I site of a pUC18m vector [50].

### Carotenoid pigment analysis

The carotenoid production level (Fig. 2) was analyzed as previously described [51] with a slight modification. Plasmids (TPS genes on the pUC vector and carotenoid genes on the pAC vector) were transformed into XL1-Blue cells, and the transformants were plated onto LB-Lennox (carb/cm) agar plates to form colonies. These colonies were picked and inoculated into 500 μL of LB-Lennox (carb/cm) medium in a 96 deep-well plate and cultured at 37°C, 1000 rpm, for 16 h. An aliquot (20 μL) of these pre-cultures was transferred to 2 mL of Terrific broth (TB) (carb/cm) in a 48 deep-well plate and cultured at 30°C, 1000 rpm for 48 h. The cells were harvested, washed with saline, and centrifuged to obtain cell pellets and the supernatants were discarded. After briefly vortexing the cell pellets, 0.5–1 mL of acetone was added to each of the pellets, and they were immediately vortexed for 1 min to extract the carotenoids, followed by centrifugation. The absorbance spectra (350–650 nm at 5-nm intervals) were analyzed for acetone extracts by using a SpectraMax Plus Absorbance Microplate Reader (Molecular Devices, Sunnyvale, CA). The pigmentation level of each culture was determined from the lambda max of the resulting extract by using the molar adsorption coefficients of carotenoids; for the C_30_ carotenoids, the diaponeurosporene constant (470 nm, 147,000 M^−1^cm^−1^), and for C_40_ carotenoids, the lycopene constant (475 nm, 185,000 M^−1^cm^−1^) were used.

### Colony screening for terpene synthase activity

*E. coli* XL1-Blue chemically competent cells harboring pAC-MN or pAC-EBI [hereafter called XL1-Blue (pAC-MN) or XL1-Blue (pAC-EBI), respectively] were prepared by using a Z-Competent *E. coli* Transformation Kit and Buffer Set (Zymo Research, CA). The TPS of interest in pUC vectors (pUC-TXS, pUC-TEAS, pUC-GES, pUC-SQS, pUC-FDS and its variants) were introduced into XL1-Blue (pAC-MN) or XL1-Blue (pAC-EBI) cells, and the resulting colonies were plated onto LB-Lennox agar containing 50 μg/mL carbenicillin (carb) and 30 μg/mL chloramphenicol (cm) topped with nitrocellulose membranes (BioTrace NT Nitrocellulose Transfer Membrane, Pall Corporation, NY). To provide uniformly spread colonies, the transformants were diluted to provide 300–500 cells/plate, and the excess amount (0.8–1 mL) of LB medium or saline was also spread on the agar plate without using a spreader, and the plates were dried. The plates were incubated at 37°C for 20–24 h to form colonies. For the cells harboring pUCara-hTXS and variants, the colony-forming nitrocellulose membrane was transferred to LB-agar (carb/cm) containing 0.2% (w/v) arabinose to induce gene expression. The colonies were incubated at room temperature (approximately 25°C) for an additional 24–60 h for pigment formation.

### Library creation and TXS screening

Using 1 pg or 10 pg of pUCara-hTXS-M60 as a template, a random mutation was inserted from the 2010^th^ to 2313^th^ nucleotides (from residues 671 to 771) of the TXS gene through error-prone PCR by adding 100 μM Mn^2+^ to the typical PCR reaction with *Taq* polymerase and the following primers: forward 5′- gctaGCTCTTCacaacgacgtcgttaaagttcaggga-3′, and reverse 5′-ctagGCTCTTCatttcatatagcaggcaattccagatgc-3′ (LguI restriction site capitalized). We ligated the resulting PCR fragment into the vector, which was amplified by using pUCara-hTXS-M60 as a template and the following primers: forward 5′-gctaGCTCTTCaaaagacaatccgggtgccac-3′, and reverse 5′-ctagGCTCTTCattgttgacttcttccatcagtttaaaccac-3′ by the FASTR cloning method [49]. The ligated product was introduced into chemically competent *E. coli* XL1-Blue cells harboring pAC-LYC. The transformants were plated onto LB-agar (carb/cm, 100–200 colonies per plate) topped with a nitrocellulose membrane. After incubating at 37°C for 20 h, the nitrocellulose membrane (with colonies) was transferred onto fresh LB-agar (carb/cm) containing 0.2% (w/v) arabinose. The cells were additionally incubated at room temperature (25°C) for 4 days, and the colonies were divided into two groups, namely beige and red clones.

The randomized regions of selected clones were subjected to colony PCR using the following primers: forward 5′-atccctgagtgcatgcagacgt-3′ and reverse 5′-gttggacggtttgaaatattcgaagga-3′. The remaining dNTPs in the PCR-amplified sample were diphosphorylated using ExoSAP-IT (GE Healthcare, Little Chalfont, UK), and the sequence was analyzed using a BigDye Terminator v3.1 Cycle Sequencing Kit (Applied Biosystems, CA) and an ABI 3130 Genetic Analyzer (Applied Biosystems, CA).

### Library creation and image analysis of the TEAS library

Random mutations were introduced into the TEAS gene by error-prone PCR. Approximately 50 ng of template DNA (pUC-TEAS) was used. Mn^2+^ was added to final concentrations of 10 or 50 μM and PCR amplified for 25 cycles using *Taq* polymerase (NEB). The PCR libraries were cloned into the *Hin*dIII/*Xho*I site of the pUC18Nm vector. The pUC-TEAS library was transformed into chemically competent XL1-Blue cells harboring pAC-MN and plated onto LB-agar topped with nitrocellulose membranes. After incubating at 37°C for 24 h to form colonies, the plates were additionally incubated at room temperature (20–25°C) for 24 h. The LB-agar plates were scanned by using a tabletop scanner (200 dpi, 24 bit RGB color). The blue channel (8 bit) of the RGB image was used to score the yellow color.

### Color screening of the TEAS library

The pUC-TEAS library was transformed into chemically competent XL1-Blue cells harboring pAC-MN as described in the previous section. Approximately 2000 colonies from two libraries with different mutation rates (an Mn^2+^ concentration of 10 or 50 μM) were screened for white colonies. Forty-two colonies (25 or 17 from TEAS-10 or TEAS-50 libraries, respectively) that exhibited a whiter color than the wild-type (parental) TEAS were cultured, and they were spotted onto LB-agar topped with nitrocellulose and incubated at 37°C for the re-screening. Finally, the plasmids were collected from 30 clones that clearly had a whiter color than the wild type.

### Product analysis of the GES

pUC-GES variants were transformed into XL1-Blue harboring pBBRSOE6 [52]. The colonies were inoculated into 500 μL of LB-Lennox (kan/carb) medium containing 0.2% glucose to suppress the leaky expression of GES in a 96-well deep well plate and cultured at 37°C, 1000 rpm for 16 h. An aliquot (100 μL) of these pre-cultures was transferred to 5 mL of TB (kan/carb) medium in a 50 mL test tube and cultured at 37°C, 200 rpm until the OD_600_ reached 0.4–0.6, and then 0.2% (w/v) arabinose and 0.1 mM IPTG were added to induce the genes, and the mixture was cultured for an additional 8 h. An aliquot (1.5 mL) of the culture was collected in 2 mL tubes and 300 μL of ethyl acetate spiked with an internal standard of (S)-(-)-limonene (Sigma-Aldrich, St. Louis, MO) was added and vortexed for 20 sec. After a short centrifugation, the organic phase was collected and analyzed by GC-FID (Shimadzu GC-2014, Shimadzu Corporation, Kyoto) equipped with an Rtx5-ms capillary column (30 m×0.25 μm ID and 0.25 μm film thickness, Restek). Splitless injections (1 μL) were performed with an injector and an FID detector temperature of 250°C, and separated with a GC oven temperature program starting at 60°C for 3 min, which was increased by 6°C min^−1^ up to 150°C, followed by an increase of 15°C min^−1^ until 230°C. For quantification, the calibration curve was drawn with a geraniol standard purchased from Sigma-Aldrich.

### Product analysis of TEAS

pUC-hTEAS and its variants were transformed into XL1-Blue cells harboring pAC-*fds-idi*, and the colonies were inoculated into 500 μL of LB-Lennox (carb/cm) medium containing 0.2% glucose in 96-well deep well plate, and cultured at 37°C, 1000 rpm for 16 h. An aliquot (100 μL) of the pre-culture was transferred to 10 mL TB (carb/cm) medium with 2% (v/v) glycerol in 50 mL test tube and cultured at 37°C, 200 rpm until the OD_600_ reached 0.4–0.6. The cultures were overlaid with 10% (v/v) dodecane (Nacalai Tesque, Kyoto), and they were shaken for an additional 48 h at 25°C, 200 rpm. Five microliters of dodecane overlay was sampled and diluted into 495 μL of ethyl acetate spiked with limonene as an internal standard. The GC-FID analysis of 5EA production was performed as described above for geraniol production analysis, except for the GC oven temperature program as follows: 60°C for 2 min followed by an increase of 20°C min^−1^ up to 230°C. Because 5EA was not commercially available, 5EA production was quantified using a calibration curve with a caryophyllene standard (TCI, Tokyo, Japan).

### Product analysis of TXS

pUC-TXS-M60 variants and pAC-CrtE were co-transformed into XL1-Blue cells, and the colonies were inoculated into 500 μL LB-Lennox (carb/cm) medium containing 0.2% (v/v) glucose in 96-well deep well plate, and they were then cultured at 37°C, 1000 rpm for 17 h. An aliquot (300 μL) of the pre-culture was transferred to 30 mL TB (carb/cm) medium in a shake flask and shaken at 30°C, 200 rpm until the OD_600_ reached 0.4–0.6. After inducing with 0.1 μM IPTG, the culture was overlaid with 10% (v/v) dodecane, and it was then shaken for an additional 48 h. The dodecane overlay was sampled and analyzed by GC-MS (Shimadzu GC-2010, Shimadzu Corporation). Splitless injections (1 μL) were performed with an injector temperature of 320°C and separated with a GC oven temperature program starting at 100°C for 3 min and increased by 10°C min^−1^ up to 300°C. The interface and detector temperatures were set to 300°C and 200°C, respectively.

## Supporting Information

Figure S1 **Production analysis of TXS-M60 and inactive TXS-M60_D613A_.** *E. coli* XL1-Blue cells harboring pAC-*crtE* and pUC-TXS were cultured, overlaid with 10% (v/v) dodecane, and sampled after 48 h of culture for GC-MS analysis. (**a**) An extracted ion chromatogram (EIC) at m/z 122. Wild type TXS-M60 peaked at RT 15.308, and there is no peak for the TXS-M60_D613A_ variant. (**b**) The mass spectrum (MS) of the product at RT 15.308 matched with the reported MS of taxa-4(5),11(12)-diene [27].
(PDF)

Figure S2 **Product analysis of TEAS and inactive TEAS_D301A_.** *E. coli* XL1-Blue cells harboring pAC-idi and pUC-hTEAS or pUC-hTEAS_D301A_ were cultured in an overlay with 10% (v/v) dodecane for 48 h, and the product was collected, diluted into ethyl acetate, and then analyzed by GC-MS. (**a**) An extracted ion chromatogram (EIC) at m/z 105. (**b**) The mass spectrum (MS) of the product at RT 11.28 in a, which matched the previously reported spectrum of 5-*epi*-aristolochene MS [54], [55]. (**c**) Chromatographs of GC-FID. Wild-type TEAS (black line) peaked at RT 11.28, and there was no peak for the TEAS_D301A_ variant (red line).
(PDF)

Figure S3 **Product analysis of GES and inactive GES_D323A_.** *E. coli* XL1-Blue cells harboring pBBRSOE6 and pUC-GES were cultured and the products were extracted by adding ethyl acetate after 8 h of culture and analyzed by GC-FID. (**a**) A chromatogram of the commercial geraniol and farnesol standard (Sigma-Aldrich). (**b**) The wild-type GES peaked at RT 14.45 min, matching the peak of the geraniol standard in a, and the GES_D323A_ variant did not. Farnesol could not be detected in both samples. The peaks approximately 15.5 and 19 min are present in both the GES and GES_D323A_ samples, which are considered to be endogenous metabolites of *E. coli*.
(PDF)

Figure S4 **Colonies of the** ***E. coli*** **cell harboring TEAS variants without mutations in N-terminal regions.** The TEAS variants were co-expressed with pAC-MN in *E. coli* XL1-Blue cells plated on LB-agar topped with nitrocellulose membranes, and they were incubated for 2 days after the colonies were formed. To provide a better view, the scanned image of the colonies was divided by the RGB and the blue channel images are shown in grayscale. The yellower colonies have the darker (black) color, and the whiter colony has a pale (light-gray) color.
(PDF)

Figure S5 **Expression level of TPSs affects carotenoid production in** ***E. coli.*** pUC-TXS or pUC-TXS_D613A_ variants (with RBS score [42] 13000), together with pUC-3000-TXS and pUC-3000-TXS_D613A_ (RBS score [42] of 3000) were co-expressed with pAC-EBI (**a**), pAC-LYC (**b**) and pAC-MN (**c**), cultured for 48 h and the carotenoid production level was analyzed. The bars represent the average of 6 samples and the error bars indicate the standard deviation.
(PDF)

Table S1 **N-terminal sequence of TEAS variants.**
(PDF)

Table S2 **Amino acid substitutions identified in selected TEAS variants with no N-terminal mutations.**
(PDF)

Table S3 **Primers used for the construction of truncated GES variants.**
(PDF)

Text S1 (PDF)
