# Supplementary material for: A High-Throughput Colorimetric Screening Assay for Terpene Synthase Activity Based on Substrate Consumption
Source: PLoS One. 2014 Mar 28;9(3):e93317. doi: 10.1371/journal.pone.0093317 (PMC3969365; doi:10.1371/journal.pone.0093317)
Supplement: Figure S1 — Production analysis of TXS-M60 and inactive TXS-M60D613A. E. coli XL1-Blue cells harboring pAC-crtE and pUC-TXS were cultured, overlaid with 10% (v/v) dodecane, and sampled after 48 h of culture for GC-MS analysis. (a) An extracted ion chromatogram (EIC) at m/z 122. Wild type TXS-M60 peaked at RT 15.308, and there is no peak for the TXS-M60D613A variant. (b) The mass spectrum (MS) of the product at RT 15.308 matched with the reported MS of taxa-4(5),11(12)-diene [27]. (PDF) [file pone.0093317.s001.pdf]

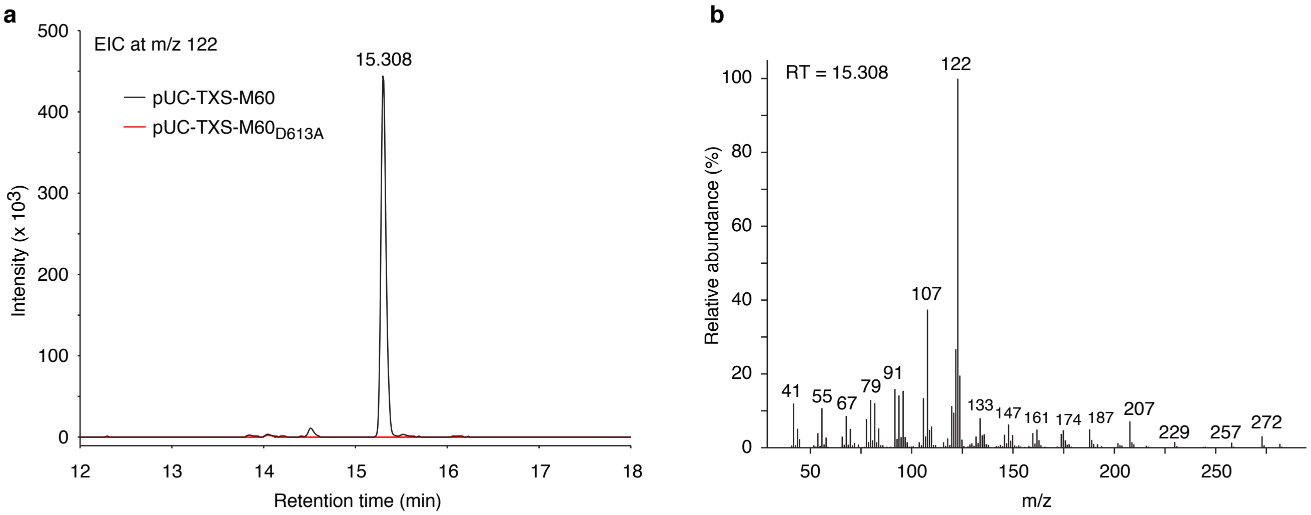

Figure S1. Production analysis of TXS-M60 and inactive TXS-M60$_{D613A}$. *E. coli* XL1-Blue cells harboring pAC-*crtE* and pUC-TXS were cultured, overlaid with 10% (v/v) dodecane, and sampled after 48 h of culture for GC-MS analysis. (a) An extracted ion chromatogram (EIC) at m/z 122. Wild type TXS-M60 peaked at RT 15.308, and there is no peak for the TXS-M60$_{D613A}$ variant. (b) The mass spectrum (MS) of the product at RT 15.308 matched with the reported MS of taxa-4(5),11(12)-diene [27].
