# Supplementary material for: A High-Throughput Colorimetric Screening Assay for Terpene Synthase Activity Based on Substrate Consumption
Source: PLoS One. 2014 Mar 28;9(3):e93317. doi: 10.1371/journal.pone.0093317 (PMC3969365; doi:10.1371/journal.pone.0093317)
Supplement: Figure S2 — Product analysis of TEAS and inactive TEASD301A. E. coli XL1-Blue cells harboring pAC-idi and pUC-hTEAS or pUC-hTEASD301A were cultured in an overlay with 10% (v/v) dodecane for 48 h, and the product was collected, diluted into ethyl acetate, and then analyzed by GC-MS. (a) An extracted ion chromatogram (EIC) at m/z 105. (b) The mass spectrum (MS) of the product at RT 11.28 in a, which matched the previously reported spectrum of 5-epi-aristolochene MS [54], [55]. (c) Chromatographs of GC-FID. Wild-type TEAS (black line) peaked at RT 11.28, and there was no peak for the TEASD301A variant (red line). (PDF) [file pone.0093317.s002.pdf]

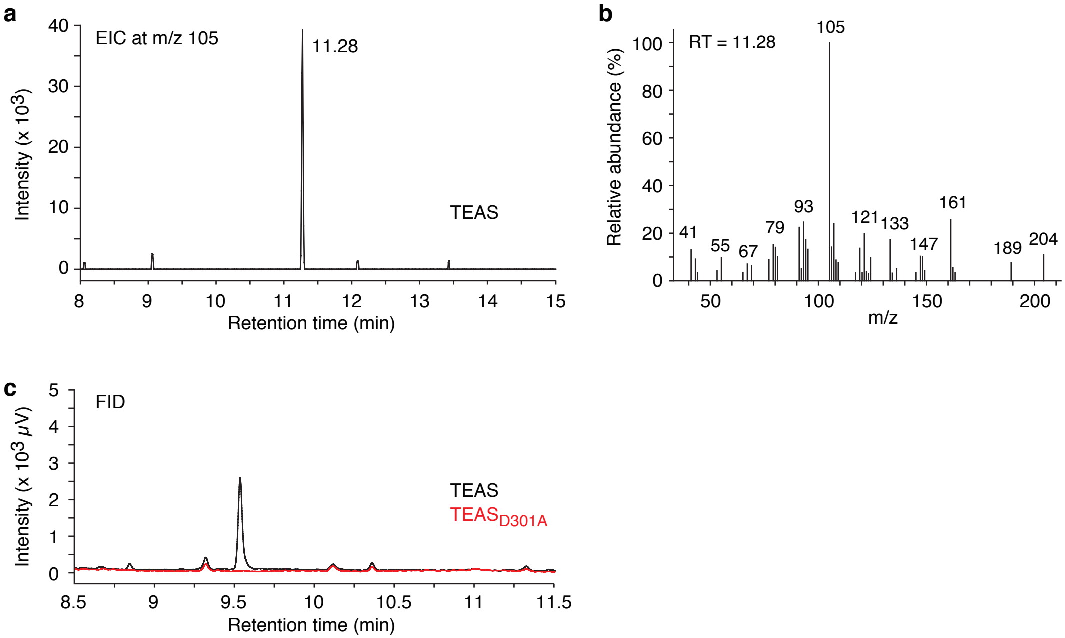

Figure S2. Product analysis of TEAS and inactive TEAS$_{D301A}$. *E. coli* XL1-Blue cells harboring pAC-idi and pUC-hTEAS or pUC-hTEAS$_{D301A}$ were cultured in an overlay with 10% (v/v) dodecane for 48 h, and the product was collected, diluted into ethyl acetate, and then analyzed by GC-MS. (a) An extracted ion chromatogram (EIC) at m/z 105. (b) The mass spectrum (MS) of the product at RT 11.28 in a, which matched the previously reported spectrum of 5-epi-aristolochene MS [54, 55]. (c) Chromatographs of GC-FID. Wild-type TEAS (black line) peaked at RT 11.28, and there was no peak for the TEAS$_{D301A}$ variant (red line).
