# Supplementary material for: A High-Throughput Colorimetric Screening Assay for Terpene Synthase Activity Based on Substrate Consumption
Source: PLoS One. 2014 Mar 28;9(3):e93317. doi: 10.1371/journal.pone.0093317 (PMC3969365; doi:10.1371/journal.pone.0093317)
Supplement: Figure S3 — Product analysis of GES and inactive GESD323A. E. coli XL1-Blue cells harboring pBBRSOE6 and pUC-GES were cultured and the products were extracted by adding ethyl acetate after 8 h of culture and analyzed by GC-FID. (a) A chromatogram of the commercial geraniol and farnesol standard (Sigma-Aldrich). (b) The wild-type GES peaked at RT 14.45 min, matching the peak of the geraniol standard in a, and the GESD323A variant did not. Farnesol could not be detected in both samples. The peaks approximately 15.5 and 19 min are present in both the GES and GESD323A samples, which are considered to be endogenous metabolites of E. coli. (PDF) [file pone.0093317.s003.pdf]

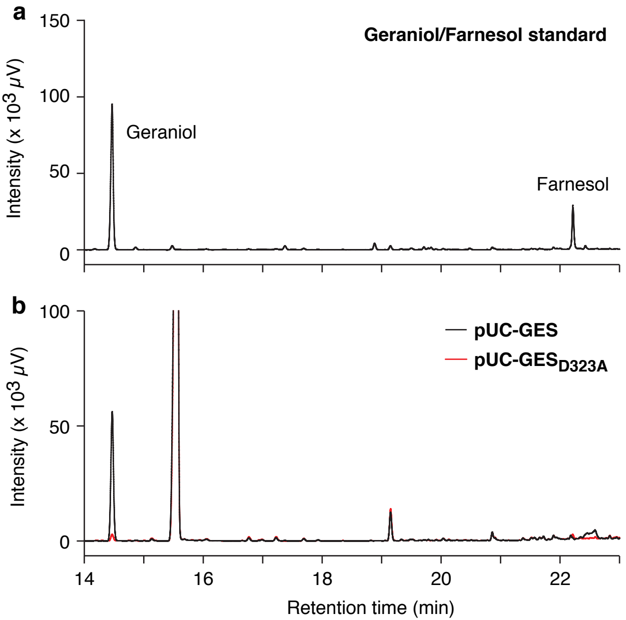

Figure S3. Product analysis of GES and inactive GES$_{D323A}$. *E. coli* XL1-Blue cells harboring pBBRSOE6 and pUC-GES were cultured and the products were extracted by adding ethyl acetate after 8 h of culture and analyzed by GC-FID. (a) A chromatogram of the commercial geraniol and farnesol standard (Sigma-Aldrich). (b) The wild-type GES peaked at RT 14.45 min, matching the peak of the geraniol standard in a, and the GES$_{D323A}$ variant did not. Farnesol could not be detected in both samples. The peaks approximately 15.5 and 19 min are present in both the GES and GES$_{D323A}$ samples, which are considered to be endogenous metabolites of *E. coli*.
