# Supplementary material for: A High-Throughput Colorimetric Screening Assay for Terpene Synthase Activity Based on Substrate Consumption
Source: PLoS One. 2014 Mar 28;9(3):e93317. doi: 10.1371/journal.pone.0093317 (PMC3969365; doi:10.1371/journal.pone.0093317)
Supplement: Figure S4 — Colonies of the E. coli cell harboring TEAS variants without mutations in N-terminal regions. The TEAS variants were co-expressed with pAC-MN in E. coli XL1-Blue cells plated on LB-agar topped with nitrocellulose membranes, and they were incubated for 2 days after the colonies were formed. To provide a better view, the scanned image of the colonies was divided by the RGB and the blue channel images are shown in grayscale. The yellower colonies have the darker (black) color, and the whiter colony has a pale (light-gray) color. (PDF) [file pone.0093317.s004.pdf]

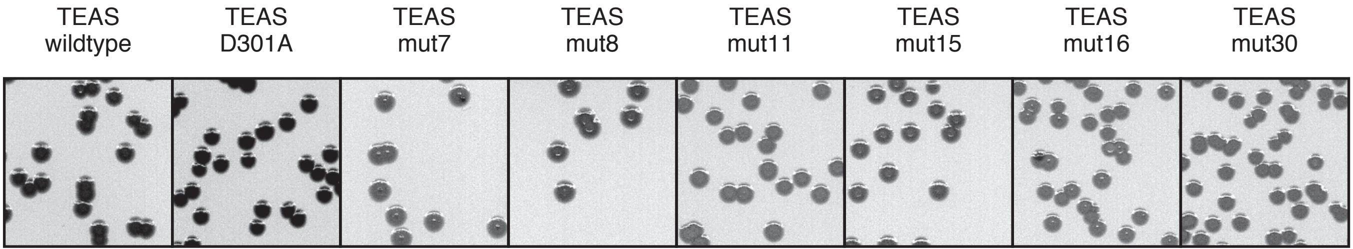

Figure S4. Colony of TEAS variants with no N-terminal mutations. The TEAS variants were co-expressed with pAC-MN in *E. coli* XL1-Blue cells plated on LB-agar topped with nitrocellulose membranes, and they were incubated for 2 days after the colonies were formed. To provide a better view, the scanned image of the colonies was divided by the RGB and the blue channel images are shown in grayscale. The yellower colonies have the darker (black) color, and the whiter colony has a pale (light-gray) color.
