# Supplementary material for: A High-Throughput Colorimetric Screening Assay for Terpene Synthase Activity Based on Substrate Consumption
Source: PLoS One. 2014 Mar 28;9(3):e93317. doi: 10.1371/journal.pone.0093317 (PMC3969365; doi:10.1371/journal.pone.0093317)
Supplement: Figure S5 — Expression level of TPSs affects carotenoid production in E. coli. pUC-TXS or pUC-TXSD613A variants (with RBS score [42] 13000), together with pUC-3000-TXS and pUC-3000-TXSD613A (RBS score [42] of 3000) were co-expressed with pAC-EBI (a), pAC-LYC (b) and pAC-MN (c), cultured for 48 h and the carotenoid production level was analyzed. The bars represent the average of 6 samples and the error bars indicate the standard deviation. (PDF) [file pone.0093317.s005.pdf]

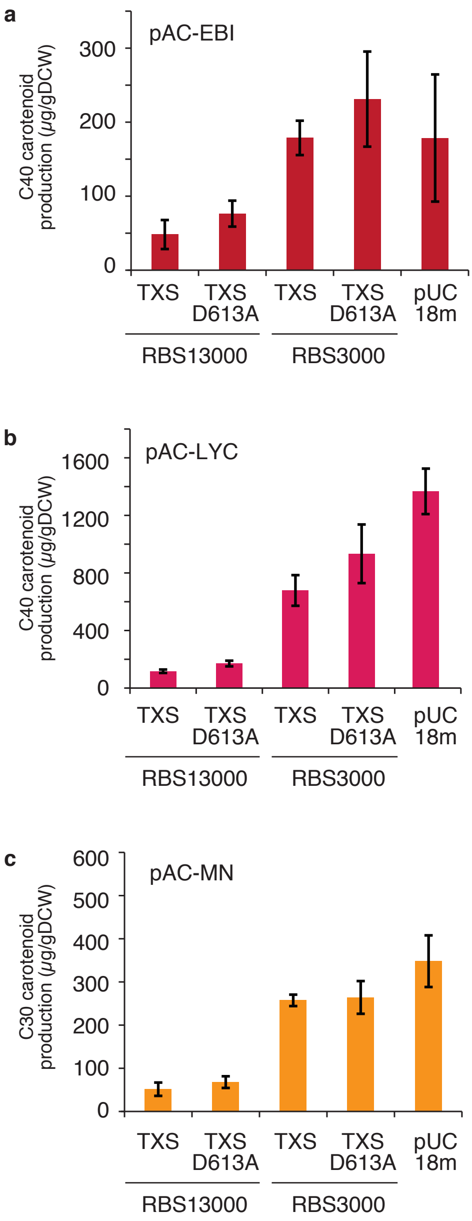

Figure S5. Expression level effects on carotenoid production. pUC-TXS or pUC-TXS_{D613A} variants (with RBS score [42] 13000), together with pUC-3000-TXS and pUC-3000-TXS_{D613A} (RBS score [42] of 3000) were co-expressed with pAC-EBI (a), pAC-LYC (b) and pAC-MN (c), cultured for 48 h and the carotenoid production level was analyzed. The bars represent the average of 6 samples and the error bars indicate the standard deviation.
