# Supplementary material for: A High-Throughput Colorimetric Screening Assay for Terpene Synthase Activity Based on Substrate Consumption
Source: PLoS One. 2014 Mar 28;9(3):e93317. doi: 10.1371/journal.pone.0093317 (PMC3969365; doi:10.1371/journal.pone.0093317)
Supplement: Table S1 — N-terminal sequence of TEAS variants. (PDF) [file pone.0093317.s006.pdf]

Table S1. N-terminal sequence of TEAS variants.

No.	Name	RBS Score[1] x 10³	Fold Increase[2]	N-terminal 35 nucleotide sequence[3]
-	**TEAS WT**	**2.0**	-	ATGCATCATCATCATCATCATGGCGCATCAGCAGC
1	TEAS mut1	9.3	4.7	ATGCATCATCATCATCATCATAGCGCATCAGCAGC
2	TEAS mut2	5.5	2.8	ATGCATTATCATCATCATCATGGCGCATCAGCAGC
3	TEAS mut3	7.3	3.7	ATGAATCATCATCATCATCATGGCGCATCAGCAGC
4	TEAS mut4	3.0	1.5	ATGTATCATCATCATCATCATGGCGCATCAGCAGC
5	TEAS mut5	6.9	3.5	ATGCATCATCATCATCATCATGGCACATCAGCAGC
6	TEAS mut6	2.3	1.2	ATGCATCATCAACATCATCATGGCGCATCAGCAGC
7	**TEAS mut7**	**2.0**	**1.0**	**ATGCATCATCATCATCATCATGGCGCATCAGCAGC**
8	**TEAS mut8**	**2.0**	**1.0**	**ATGCATCATCATCATCATCATGGCGCATCAGCAGC**
9	TEAS mut9	5.5	2.8	ATGCATTATCATCATCATCATGGCGCATCAGCAGC
10	TEAS mut10	6.9	3.5	ATGCATAATCATCATCATCATGGCGCATCAGCAGC
11	**TEAS mut11**	**2.0**	**1.0**	**ATGCATCATCATCATCATCATGGCGCATCAGCAGC**
12	TEAS mut12	6.2	3.1	ATGCATCATCATCATCATCAGGGCGCATCAGCAGC
13	TEAS mut13	3.0	1.5	ATGCATCATCATCATCATCATGGCGCACCAACAGC
14	TEAS mut14	3.0	1.5	ATGTATCATCATCATCATCATGGCGCATCAGCAGC
15	**TEAS mut15**	**2.0**	**1.0**	**ATGCATCATCATCATCATCATGGCGCATCAGCAGC**
16	**TEAS mut16**	**2.0**	**1.0**	**ATGCATCATCATCATCATCATGGCGCATCAGCAGC**
17	TEAS mut17	6.0	3.0	ATGCATCATCATCATCATCAAGGCGCATCAGCAGC
18	TEAS mut18	3.0	1.5	ATGTATCATCATCATCATCATGGCGCATCAGCAGC
19	TEAS mut19	6.0	3.0	ATGCATCATCATCATCATCACGGCGCATCAGCAGC
20	TEAS mut20	3.2	1.6	ATGCAGCATCATCATCATCATGGCGCATCAGCAGC
21	TEAS mut21	14.9	7.5	ATGCATCATCATCATCAT---GGCGCACCAGCAGC
22	TEAS mut22	2.8	1.4	ATGCATCATCATCATCATCATGGCGCACCAGCAGC
23	TEAS mut23	8.0	4.0	ATGCATCATCATCATCATCATGCCGCATCAGCAGC
24	TEAS mut24	2.8	1.4	ATGCATCATCATCATCATCATGGCGCACCAGCAGC
25	**TEAS mut25**	**2.0**	**1.0**	**ATGCATCATCATCATCATCATGGCGCATCAGCAGC**
26	TEAS mut26	3.0	1.5	ATGCATCATCATCATCATCATGGCGTATCAGCAGC
27	TEAS mut27	5.1	2.6	ATGCATCATCATCATCATCATGGCGCCACAGCAGC
28	TEAS mut28	2.8	1.4	ATGCATCATCATCATCATCATGGCGCACCAGCAGC
29	TEAS mut29	6.9	3.5	ATGCATCATCATCATCATCATGGCCCATCAGCAGC
30	**TEAS mut30**	**2.0**	**1.0**	**ATGCATCATCATCATCATCATGGCGCATCAGCAGC**

The TEAS variants with no N-terminal 35 nt substitution are indicated in boldface.

[1]The RBS score was calculated by an RBS calculator.

[2]The fold increase of the RBS score was compared with the TEAS WT as shown.

[3]The N-terminal 35 nt of the ORF together with the 35 nt of 5’-UTR sequence (5’-attgtgagcggataacaatttcacacgaattctctagaAGGAGGaagctta-3’) was used as a query sequence for the RBS calculator. The mutated nucleotides are indicated in red and deleted nucleotides are indicated with a hyphen (-).
