# Supplementary material for: A High-Throughput Colorimetric Screening Assay for Terpene Synthase Activity Based on Substrate Consumption
Source: PLoS One. 2014 Mar 28;9(3):e93317. doi: 10.1371/journal.pone.0093317 (PMC3969365; doi:10.1371/journal.pone.0093317)
Supplement: Table S2 — Amino acid substitutions identified in selected TEAS variants with no N-terminal mutations. (PDF) [file pone.0093317.s007.pdf]

Table S2. Amino acid substitutions identified in selected TEAS variants with no N-terminal mutations.

Name	Nucleotide mutations (amino acid substitutions in parentheses)
TEAS mut7	A1442G (**Q481R**), A1605G
TEAS mut8	T369C, A1421G (**K474R**)
TEAS mut11	T1196G (**L399R**), T1503C, C1638T
TEAS mut15	A1257T, A1543T (**I515V**), A1615G (**I539V**)
TEAS mut16	A456G, A1442G (**Q481R**), A1519T (**T507S**)
TEAS mut25	T46C (**Y16H**), T126C (**T313C**), G549A (**C105R**), A1019G (**K340R**)
TEAS mut30	A514T (**T172S**), G525A, T1159A (**Y387N**)
