# Supplementary material for: A High-Throughput Colorimetric Screening Assay for Terpene Synthase Activity Based on Substrate Consumption
Source: PLoS One. 2014 Mar 28;9(3):e93317. doi: 10.1371/journal.pone.0093317 (PMC3969365; doi:10.1371/journal.pone.0093317)
Supplement: Table S3 — Primers used for the construction of truncated GES variants. (PDF) [file pone.0093317.s008.pdf]

Table S3. Primers used for the construction of truncated GES variants.

Primer Name	Sequence[1]
GEStrunc-R	CTAG**GCTCTTC**TCATTTGTAATCCTCCTGAATTCCATATGTGTTTCCT
M44-F	GCTA**GCTCTTC**AATGCCTCTAAGTTCAACTCCTCTCATCAAC
M47-F	GCTA**GCTCTTC**AATGTCAACTCCTCTCATCAACGGGGATA
M49-F	GCTA**GCTCTTC**AATGCCTCTCATCAACGGGGATAACTCTCA
M53-F	GCTA**GCTCTTC**AATGGGGGATAACTCTCAGCGTAAAAACACAC
M60-F	GCTA**GCTCTTC**AATGAACACACGTCAACACATGGAGGAGAGCA
M66-F	GCTA**GCTCTTC**AATGGAGGAGAGCAGCAGCAAGAG
M69-F	GCTA**GCTCTTC**AATGAGCAGCAAGAGGAGAGAATATCTGCTGGA
M77-F	GCTA**GCTCTTC**AATGCTGGAGGAAACGACGCGAAAACT
M99-F	GCTA**GCTCTTC**AATGGACAACATCCAACAGTTGGGAATCGGCTACTATTTT

[1]The LguI restriction recognition site is indicated by boldface, and the digestion site (5’ -overhang) is underlined.
