# Supplementary material for: A High-Throughput Colorimetric Screening Assay for Terpene Synthase Activity Based on Substrate Consumption
Source: PLoS One. 2014 Mar 28;9(3):e93317. doi: 10.1371/journal.pone.0093317 (PMC3969365; doi:10.1371/journal.pone.0093317)
Supplement: Text S1 — (PDF) [file pone.0093317.s009.pdf]

Supporting information Text S1 for:

A high-throughput colorimetric screening assay for terpene synthase activity based on substrate consumption

Maiko Furubayashi, Mayu Ikezumi, Jun Kajiwara, Miki Iwasaki, Akira Fujii, Ling Li, Kyoichi Saito and Daisuke Umeno*

---

1. Notes for screening-system setup

1.1. Expression balancing

The over-expression of TPS could decrease pigment production without activity: We found that even the inactivated TPS variants could lead to color fading in the cells harboring carotenoid pathways, if its expression level was high (**Fig. S5**). This result is most likely explained by the depletion of translation resources (aminoacyl-tRNAs and ribosome), resulting in a decreased expression level of CrtM/CrtN. In the system presented in this paper, TPS is expressed under the control of a *lac* promoter (without induction). In this particular construct, this effect is negligible as long as the RBS scores [1] for the TPS gene remain below 3000.

Excessively high TPSs/carotenoid gene expression could be toxic to the cell: We found that the use of excessively strong promoters/RBSs for these enzymes dramatically reduces the growth rate, and in some cases, their expression completely abolishes cell viability, most likely because of the depleted transcriptional/translational resources. For example, the leaky expression of CrtM and CrtN under the *lac* promoter without a *lac* operator on p15A vector, and the co-expression of the GES gene under the control of the *lac* promoter (with operator) on the pMB1 vector, failed to form colonies on LB-agar. This finding is somewhat consistent with previous reports in which the elevated induction level of carotenoid genes frequently resulted in decreased cell growth and carotenoid production (for example, [2]).

1.2. A precursor feeding enzyme can be used to adjust the screening threshold level

When the cellular activity of the TPS of interest is high enough, it could virtually dominate the substrates within the cell. This activity could cause the complete loss of carotenoid pigmentation (for instance, see FDS variants Fig. 2b). In this situation, it is impossible to search for variants with improved activity. Reducing the expression level of TPS (discussed

above at section 1.1) is one good solution to solve this challenge: one should tune the promoter strength or translation efficiency down to the level in which the wild type (parent for evolution) yields some level of pigmentation. However, this solution has one downside; by down-regulate the expression of TPSs, one could significantly increase the chance of bouncing back in TPS expression.

Screening threshold could be also tuned by changing the precursor availability. The additional expression of precursor enzymes and the feeding capacity of the precursor could be significantly increased. Above the certain level of substrate, even the highly active TPSs (or their variants) could partially leave the precursor for carotenoid synthesis, thereby allowing carotenoid genes to produce pigments. We found that a slight increase in the precursor feeding capacity, for instance by the additional expression of Idi, is enough to possibly set up a screening system for evolving TPSs that shows already high activity. For example, we used pAC-MN-idi for the directed evolution of TEAS (Fig. 5b), since the wildtype TEAS did not produce enough carotenoids pigment when co-expressed with pAC-MN (without idi).

2. pAC-MN plasmid sequence

During the early stage of this work, we constructed several prototype plasmids for expressing CrtM and CrtN, in which we found some instability in the pigmentation. We happened to acquire a very stable and reproducible screening plasmid carrying the *E. coli* insertion sequence (IS10) [3] between the *lac* promoter and *crtM* gene. When the IS10 sequence was removed, the plasmids could not be co-transformed with TPS genes to *E. coli* cells, strongly indicating that the instability came from the metabolic burden to the host cell because of the unnecessarily high expression of carotenoid enzymes. Therefore, we decided to adopt the plasmid containing the IS10 sequence. The pAC-MN sequence is shown below.

The sequences indicated below (flanked by *Bam*HI) was cloned into the *Bam*HI restriction site of pACmod. *lac* promoter is underlined, and *Bam*HI site (GGATCC) is in boldface. Letters in red corresponds to IS10 sequence, while *crtM/crtN* genes are indicated in blue.

GGATCCaggtttcccgactggaaagcgggcagtgagcgcaacgcaattaatgtgagttagctcactcattaggca
ccccaggctttacactttatgcttccggctcgtAtgttgtgtggaattgtgagcgtctagagaacaataantcna
ggtaatggcatttcatataggaggACTAGTatgacaatgatgGATActgagagatcccctcataatttccccaaa
acgtaaccatgtgtgaatagattttgagtaagcagggttgcagccacgagtgagtcttcccttgttattgtgtag
ccagaatgccgcaaaacttccatgcctaagcgaactgttgagagtacgtttcgatttctgactgtgttagcctgg
aagtgcttgtcccaaccttgtttctgagcatgaacgcccgcaagccaacatgttagttgaagcatcagggcgatt
agcagcatgatatcaaaacgctctgagctgctcgttcggctatggcgtaggcctagtccgtaggcaggactttc
aagtctcggaaggtttcttcaatctgcattcgcttcgaatagatattaacaagttgtttgggtgttcgaatttca
acaggtaagttagttgctagaatccatggctcctttgccgacgctgagtagattttaggtgacgggtggtgacaa
tgagtccgtgtcgagcgctgatttttcggcctttagagcgagatttatacaatagaatttggcatgagattgga
ttgcttttagtcagcctcttatagcctaaagtctttgagtgactagatgacatatcatgtaagttgctgataggt
ttccagttttccgctcctaggtctgcatattgtacttttcctcttactcgacttaaccagtaccaacccagcttc
tcaacggatttataccatggcactttaaagccagcatcactgacaatgagcggtgtggtgttactcggtagaatg
ctcgcaaggtcggctagaaattggtcatgagctttctttgaacattgctctgaaagcgggaacgctttctcataa
agagtaacagaacgaccgtgtagtgcgactgaagctcgcaataccataagccgtttttgctcacggatatcagac
cagtcaacaagtacaatgggcatcgtattgcccgaacagataaagctagcatgccaacggtatacagcgagtcgc
tctttgtggaggtgacgattacctaacaatcggtcgattcgtttgatgttatgttttgttctcgctttggttggc
aggttacggccaagttcggtaagagtgagagttttacagtcaagtaaggcgtggcaagccaacgttaagctgttg
agtcgttttaagtgtaattcggggcagaattggtaaagagagtcgtgtaaaatatcgagttcgcacattttgttg
tctgattattgatttttggcgaaaccatttgatcatatgacaagatgtgtatctaccttaacttaatgattttga

taaaaatcattaggggattcatcagTGatggatatgaattttaaatattgtcataaaatcatgaagaaacattca
aaaagcttttcttacgcttttgacttgttaccagaagatcaaagaaaagcggtttgggcaatttatgctgtgtgt
cgtaaaattgatgacagtatagatgtttatggcgatattcaatttttaaatcaaataaaagaagatatacaatct
attgaaaaatacccatatgaacatcatcactttcaaagtgatcgtagaatcatgatggcgcttcagcatgttgca
caacataaaaatatcgcctttcaatctttttataatctcattgatactgtatataaagatcaacattttacaatg
tttgaaacggacgctgaattattcggatattgttatggtgttgctggtacagtaggtgaagtattgacgccgatt
ttaagtgatcatgaaacacatcagacatacgatgtcgcaagaagacttggtgaatcgttgcaattgattaatata
ttaagagatgtcggtgaagattttgacaatgaacggatatattttagtaagcaacgattaaagcaatatgaagtt
gatattgctgaagtgtaccaaaatggtgttaataatcattatattgacttatgggaatattatgcagctatcgca
gaaaaagattttcaagatgttatggatcaaatcaaagtatttagtattgaagcacaaccaatcatagaattagca
gcacgtatatatattgaaatactggacgaagtgagacaggctaactatacattacatgaacgtgtttttgtggat
aagaggaaaaaggcaaagttgtttcatgaaataaatagtaaatatcatagaatatagGTGGTTGAATAatgaaga
ttgcagtaattggtgcaggtgtcacaggattagcagcggcagcccgtattgcttctcaaggtcatgaagtgacga
tatttgaaaaaaataataatgtaggcgggcgtatgaatcaattaaagaaagacggctttacatttgatatgggtc
ccacaattgtcatgatgccagatgtttataaagatgtttttacagcgtgtggtaaaaattatgaagattatattg
aattgagacaattacgttatatttacgatgtgtattttgaccacgatgatcgtataacggtgcctacagatttag
ctgaattacagcaaatgctagaaagtatagaacctggttcaacgcatggttttatgtccttttaacggatgttt
ataaaaaatatgaaattgcacgtcgctatttcttagaaagaacgtatcgcaaaccgagtgacttttataatatga
cgtcacttgtgcaaggtgctaagttaaaaacgttaaatcatgcagatcagctaattgaacattatattgataacg
aaaagatacaaaagcttttagcgtttcaaacgttatacataggaattgatccaaaacgaggcccgtcactatatt
caattattcctatgattgaaatgatgtttggtgtgcattttattaaaggcggtatgtatggcatggctcaagggc
tagcgcaattaaataaagacttaggcgttaatattgaactaaatgctgaaattgagcaaattattattgatccta
aattcaaacgggccgatgcgataaaagtgaatggtgacataagaaaatttgataaaattttatgtacggctgatt
tccctagtgttgcggaatcattaatgccagattttgcacctattaaaaagtatccaccacataaaattgcagact
tagattactcttgttcagcattttaatgtatatcggtatagatattgatgtgacagatcaagtgagacttcata
atgttatttttcagatgactttagaggcaatattgaagaaatatttgagggacgtttatcatatgatccttcta
tttatgtgtatgtaccagcggtcgctgataaatcacttgcgccagaaggcaaaactggtatttatgtgctaatgc
cgacgccggaacttaaaacaggtagcggaatcgattggtcagatgaagctttgacgcaacaaataaaggaaatta
tttatcgtaaattagcaacgattgaagtatttgaagatataaaatcgcatattgtttcagaaacaatctttacgc
caaatgattttgagcaaacgtatcatgcgaaatttggttcggcattcggtttaatgccaactttagcgcaaagta
attattatcgtccacaaaatgtatcgcgagattataaagatttatattttgcaggtgcaagtacgcatccaggtg
caggcgttcctattgtcttaacgagtgcgaaaataactgtagatgaaatgattaaagatattgagcggggcgtat
aaGAATTCCCATGGGCGGCCGCtgcggtattttctccttacgcatctgtgcggtatttcac**GGATCC**

References

1. Salis HM (2011) The ribosome binding site calculator. Methods Enzymol 498: 19-42.
2. Yoon SH, Kim JE, Lee SH, Park HM, Choi MS, et al. (2007) Engineering the lycopene synthetic pathway in *E. coli* by comparison of the carotenoid genes of *Pantoea agglomerans* and *Pantoea ananatis*. Appl Microbiol Biotechnol 74: 131-139.
3. Kovarik A, Matzke MA, Matzke AJ and Koulakova B (2001) Transposition of IS10 from the host *Escherichia coli* genome to a plasmid may lead to cloning artefacts. Mol Genet Genomics 266: 216-222.
